# Fistula Lachrymalis and Mylopetroleum Ointment

**Published:** 1882-08

**Authors:** C. F. Nichols

**Affiliations:** Boston; Bureau of Materia Medica, etc., I. H. A.


### CLINICAL BUREAU.

---

### FISTULA LACHRYMALIS AND MYLOPETROLEUM OINTMENT.

C. F. Nichols, M. D., Boston.

(*Bureau of Materia Medica, etc., I. H. A.*)

1879, February 4th, Miss M——, aged sixty, spare, of light complexion. For a sore throat, a "Professor" (!) of Homœopathy, *commencing its treatment*, applied Mylopetroleum to the neck. This patient had suffered, two years before, from an abscess in the right inner canthus, and her eyes had been weak and lachrymose since its occurrence. Her throat directly got well, but abscesses formed repeatedly above both lachrymal canals until fistulæ were established.* Much burning pain and throbbing during the filling of the sac. The discharge thick and yellow. The swelling *copper-red and erysipelatous.* Passing to the left and also remaining in the right orbit. Pain relieved by warm compresses. Patient apprehensive. No appetite. Catarrhal. Drowsy. Chilly. Weak. *Worse forenoons.*

These remedies were given in succession, from three to eight weeks apart, as changes in characteristics called for the choice of each:— Sil., Sulph., Calc. carb., Nat. phos., Graphit., Puls. (after Puls. the patient was sad, wept when looked at), Lycop., Merc. sol., Euphras.,

---

* Mylopetroleum is recommended for sore eyes, but I am not aware that provings of any of its effects have been published.

Fluor. ac., Baryta mur., Staphys. The Sil. in CM (Swan) potency and 12 cent. trit., the others CM, and DM (Swan). After Euphras. and Sil., brief amelioration.

November 9th, 1880, Petrol. CM. January 25th, 1881, Kali bi. CM, were given in consultation with Dr. J. B. Bell.

In October there seemed to be no real improvement, although the pus-filling of the lachrymal sac culminated less frequently. Both eyes were exceedingly photophobic, lachrymose, with lachrymal ducts completely closed. The left side filled more often than the right. Discharges acrid, excoriating, worse at night, in wind; inner canthi worse. Lids congested, the lower lids granulated, bluish. Vertigo. Cheerful, with turns of much depression, mostly anxious and nervous, with pale face.

October 10th. Given Zinc Dm.

February 11th, 1881. Kreos. cm was given. Petrol. had been given by Dr. Bell, with sufficient symptoms *as an analogue of Mylo-petroleum,* here was a potency from a similar product. See Hering's *Condensed Materia Medica* for symptoms.

Since Kreos., one sac (right side) has once refilled, and only once, the eyes have gradually become healthy, save unusual tear-supply when exposed to wind.

Two oculists ("qualified" and regular), friends of the patient, who twice examined the eyes (1879, 1880), diagnosed fistula and insisted on local treatment, now express astonishment at two things: The first obstinate refusal to be frightened into local treatment, and the present free and normal state of the tear passages.

